# PVA-Based Electrospun Biomembranes with Hydrolyzed Collagen and Ethanolic Extract of *Hypericum perforatum* for Potential Use as Wound Dressing: Fabrication and Characterization

**DOI:** 10.3390/polym14101981

**Published:** 2022-05-12

**Authors:** Alitzel Belém García-Hernández, Eduardo Morales-Sánchez, Blanca M. Berdeja-Martínez, Monserrat Escamilla-García, Ma. Paz Salgado-Cruz, Minerva Rentería-Ortega, Reynold R. Farrera-Rebollo, Miguel A. Vega-Cuellar, Georgina Calderón-Domínguez

**Affiliations:** 1 Escuela Nacional de Ciencias Biológicas, Instituto Politécnico Nacional, CDMX, Mexico City 07738, Mexico; agarciah1805@alumno.ipn.mx (A.B.G.-H.); bberdejam@ipn.mx (B.M.B.-M.); msalgadoc@ipn.mx (M.P.S.-C.); rfarrera@ipn.mx (R.R.F.-R.); mvegac1600@alumno.ipn.mx (M.A.V.-C.); 2 Centro de Investigación en Ciencia Aplicada y Tecnología Avanzada, Unidad Querétaro, Instituto Politécnico Nacional, Qro., Santiago de Querétaro 76090, Mexico; emoraless@ipn.mx; 3 Departamento de Investigación en Alimentos y Estudios de Postgrado, Universidad Autónoma de Querétaro, Qro., Santiago de Querétaro 76010, Mexico; monserrat.escamilla@uaq.mx; 4 Tecnológico Nacional de México/TES de San Felipe del Progreso, Edo. Méx., San Felipe del Progreso 50640, Mexico; minerva.ro@sfelipeprogreso.tecnm.mx

**Keywords:** *Hypericum perforatum*, hydrolyzed collagen, polyvinyl alcohol, electrospinning, wound dressing

## Abstract

Biological, physicochemical, structural, and thermal properties of PVA-based electrospun wound dressings added with hydrolyzed collagen (HC) and different concentrations of *Hypericum perforatum* ethanolic extract (EEHP) were studied. Membrane characterization was carried out by X-ray diffraction, Fourier infrared spectroscopy, differential scanning calorimetry, barrier properties, scanning electron microscopy, image analysis (diameter and pore size), as well as antimicrobial and anti-inflammatory activities. Results showed that the PVA/HC/EEHP materials, fabricated under controlled conditions of temperature and humidity, generated fiber membranes with diameters between 140–390 nm, adequate porosity and pore size for cell growth (67–90% and 4–16 µm, respectively), and good barrier properties (0.005–0.032 g·m^−2^ s^−1^) to be used in the treatment of conditions on the skin, and was even better than some commercial products. Finally, they showed to have anti-inflammatory (>80%), and antimicrobial activity against *S. aureus* and *S. epiderm*. Furthermore, higher crystalline structure was observed according to the EEHP concentration. In addition, this is the first report in which PVA/HC/EEHP membranes are successfully fabricated and characterized.

## 1. Introduction

Since the last decades, the biomedical field through tissue engineering, an interdisciplinary field (chemistry, physics, material science, engineering), has been challenged to produce promising functional biomedical devices to treat skin damages (e.g., scaffolds, delivery systems, or wound dressings). In the case of wound dressings, an ideal one should provide a physical barrier to protect the wound from any exogenous organisms, minimize infection and prevent excessive fluid loss but maintain a moist healing environment to avoid dehydration. It must also consider promoting epithelial restoration and having high porosity values for gases permeation, nutrients transportation, and cell adhesion, proliferation, and differentiation. It must be biocompatible (non-toxic, non-allergic) and cheap as well [1,2,3,4,5].

Due to the wound dressing’s requirements, the manufacturing process must be very specific. Indeed, some methods such as electrospinning (ES) can be used. ES is a kind, tunable, and easy to operate electrohydrodynamic technique, which permits the production of electrospun fibers with a size range from a few micrometers to tens of nanometers and with properties dependent on the ambient and processing parameters, as well as the solutions’ physicochemical properties, which together will determine the characteristics of the products [6]. This method allows obtaining many kinds of systems to be used in the biomedical field, e.g., membranes or scaffolds for tissue engineering to repair cardiac tissue, to treatment of nerve injury, for wound healing, for bone and skeletal muscle regeneration among others [7]; to fabricate biosensors [8]; to produce drug delivery systems [9] for distribution of proteins, drugs, or any bioactive compounds, etc.; to deposit non-woven micro/nanofibers which mimic the extracellular matrix, exhibiting a high surface-to-volume ratio high porosity as reported by Azimi B. et al., [10]. The principle of this process is based on the combination of the surface tension, electrostatic, and viscoelastic forces being the electrostatic repulsion the main driving force for fiber formation [11].

While ES is adjusted to obtain optimized structural and morphological materials to accomplish the requirements, the component selection also plays a crucial role. Many synthetic (PEG, PCL [12,13], PEO [14], and PVA) and natural polymers (cellulose [15], chitin [16], and collagen) have been used. From these materials, polyvinyl alcohol (PVA) is an excellent option as supporting media for wound dressings, due to it is biodegradable, non-toxic, and good fiber formation capacity that results in mats with optimal flexibility. Additionally, it is a water-soluble polymer with excellent mechanical, chemical, and thermal properties [17,18]. However, its biological properties are recommended to be improved by adding bioactive molecules from animal or plant sources. These molecules are chemicals that can contribute to a faster and more robust wound healing process. Bioactive compounds from animal origin have been extensively incorporated in wound dressings, mainly chitosan [19,20,21], collagen [22,23], and gelatin [24,25]. However, other materials such as hydrolyzed collagen (HC) promise better absorption and distribution of amino acids in the human body through the bloodstream [26] due to their lower molecular weight (0.3–8 kDa) compared with collagen and gelatin. Moreover, according to the literature, the amino acids of the HC act as systemic support, optimizing the immune response, providing the dermis with the essential components for the formation of collagen fibers when they are found as free amino acids, and acting as ligands, binding to the receptors on the fibroblast membrane and stimulating the production of new collagen when they are as oligopeptides [27].

Regarding plant bioactive molecules, better known as secondary metabolites, they are good options for wound healing due to their non-toxic features and high compatibility with physiologic and biological conditions [3,5], e.g., De la Ossa et al., (2019) have reported the biological activity of olive leaf extract and then its incorporation into polyhydroxyalkanoate fibers for use as scaffolds (2021) [28,29]. However, despite their well-reported properties, only a few groups have reported the bioactive compound incorporation to improve the biological properties of materials [30,31,32] and even less research has been done on electrospun nanofibers; therefore, the addition of these molecules as proposed in this work is relevant, knowing as well that their addition into a nanostructured device increases the bioavailability of the compounds due to the high surface-to-volume ratio of the fibers.

Commonly, *Aloe vera* [33], Agrimonia eupatoria [34], *Calendula officinalis* [35], cinnamon [36], curcumin [37], Glycyrrhuza glabra (as Glycyrrhetinic acid) [38], *Momordica charantia antimicrobial* [39], and Nepeta dschuparensis [40] have been implemented in biomedical devices such as wound dressings due to their antimicrobial, anti-inflammatory, antiviral, and antioxidant (among other well-stablished biological) properties. Nevertheless, *Hypericum perforatum*, a traditional medicinal material, has also been extensively reported as an effective antimicrobial as well as anti-inflammatory, antioxidant, and healing material due to the presence of secondary metabolites such as naphthodianthrones, flavonoids, phenolic compounds, and tannins among others, which can convert it into a potential candidate for biomedical materials [41,42,43], especially for wound dressings.

*Hypericum perforatum* extracts have previously been incorporated into based-PCL [3], PLA [44], PCL/Gelatin [45], and PEG/PCL [4] electrospun membranes. However, there is not any report on electrospun biomembranes based on PVA added with EEHP and HC. The excellent PVA chemical, thermal, and mechanical properties could play a more important role in the biomedical field in combination with EEHP and HC. This suggests that the PVA/HC/EEHP nanofibers will possess properties highly favorable for utilizing in healing material for skin diseases. In this sense, the aim of this research was to develop a biomembrane with appropriate microstructural properties and antimicrobial and anti-inflammatory activities for potential use as wound dressing. We have produced membranes based on PVA with HC and EEHP by the electrospinning technique. First, an HP extract was obtained by maceration in ethanol. The resulting extract was incorporated into a PVA and PVA/HC solutions and electrospun. Then, morphological, thermal, physical (thickness, porosity, diameter fibers), and chemical characteristics were studied. Finally, anti-inflammatory and antimicrobial activities were evaluated by in vitro techniques.

## 2. Materials and Methods

### 2.1. Materials

Polyvinyl alcohol (PVA) with *M_W_* = 146,000–186,000 g·mol^−1^ (Sigma-Aldrich, St. Louis, MO, USA). Hydrolyzed collagen (HC) was obtained from Costco^®^ (Gelita, Germany). *Hypericum perforatum* (HP) as a dried plant was acquired from REDSA (Venustiano Carranza, CDMX, Mexico).

### 2.2. Preparation of Ethanolic Extract of Hypericum perforatum L. (EEHP)

The ethanolic extract was prepared by maceration with *Hypericum perforatum* dried powder in a ratio of 1:6 g:mL in ethanol 96% (EtOH; DIBAR, Mexico). Maceration was maintained during seven days with manual agitation every 8 h. Finally, the solution was filtered with filter paper Whatman No. 1 under vacuum. It was then stored in an amber glass bottle at room temperature (~21 °C).

### 2.3. Solutions Preparation

PVA, PVA/HC, and PVA/HC/EEHP solutions were prepared at a concentration of 6% *w v*^−1^ with the report by García-Hernández et al. (2021) [46]. Briefly, PVA solutions were created by dissolving the granules in hot (at 75 °C) distilled water (DW; Reasol, Mexico) under magnetic stirring (Cimarec, Thermo Fisher Scientific, Wattham, MA, USA) at 600 rpm until complete dissolution (approximately three hours). To prepare the hydrolyzed collagen (HC) solution, the HC powder was dissolved in hot (40 °C) DW under magnetic stirring for 5 min. Hydrolyzed collagen was added at 0 and 5% *w v*^−1^, while four different concentrations (0, 8, 16, 32% (*w v*^−1^)) of ethanolic extract of *Hypericum perforatum* (EEHP) were used.

To prepare PVA, PVA/HC, and PVA/EEHP solutions, 10 mL of the PVA solution were mixed with EtOH, HC, or EEHP in a ratio of 10:1 mL (PVA:liquid: EtOH, HC, or EEHP) after complete dissolution and cooled at room temperature (~25 °C), while the three-component (PVA/HC/EEHP) solutions were prepared as follows: 10:0.5:0.5 mL of PVA:HC:EEHP. The HC and EEHP were added slowly into the PVA solution under magnetic stirring at 600 rpm at room temperature and stirred for 5 min. Table 1 shows the concentration of the three components (PVA, HC, and EEHP) used for each membrane, as well as the nomenclature to identify the samples.

### 2.4. Fabrication of Electrospun Membranes

**Figure 1:**
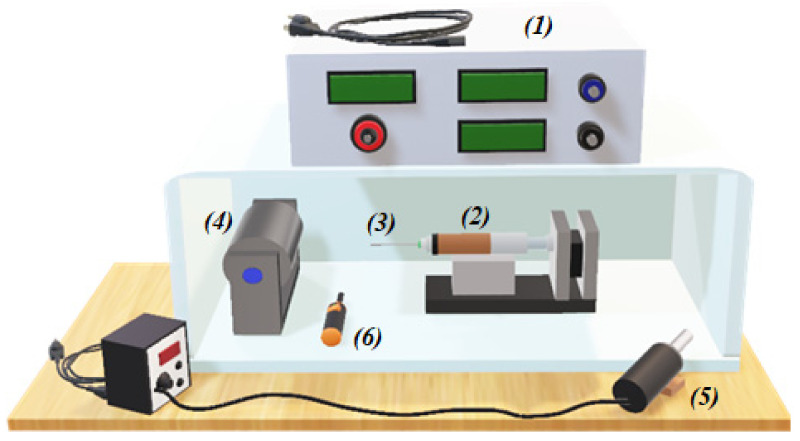
Configuration of the electrospinning device used for the fabrication of the PVA/HC/EEHP membranes: (**1**) high-voltage power supply, (**2**) syringe pump, (**3**) capillary (stainless steel needle), (**4**) rotating collector, (**5**) hot-air gun (welding station), and (**6**) thermohygrometer.

The electrospinning process was stablished in accordance with the methodology reported by García-Hernández et al. (2021) [46]. Each polymeric solution was placed in a plastic syringe equipped with a stainless steel needle (21G, 0.8192 mm), to be injected at a high voltage of 15 kV with a controllable pump (30A24-P4, UltraVolt, Inc., Ronkonkoma, NY, USA) at a flow rate of 1 mL h^−1^. PVA/HC/EEHP membranes were obtained at 10 cm from needle to collector by injecting 3 mL of each prepared solution (Table 1). A stainless steel rotating drum (9.5 × 15 × 5 cm^3^; 0.843 rpm) grounded and controlled by a stepping motor was used to collect the nanofibers. Ambient conditions were stablished at 36 ± 2% of relative humidity (RH) and 30 ± 2 °C of temperature. To maintain these conditions, a hot air welding station (Steren, CAU280, Azcapotzalco, CDMX, Mexico) was coupled to the electrospinning chamber, as well as a thermohygrometer (Testo, 605i, Titisee-Neustadt, BW, Germany) to monitor them while the injection progressed as shown in Figure 1.

### 2.5. Physicochemical Characterization of the Polymeric Solutions

The solutions’ physicochemical characterization was carried out according to their conductivity, density, viscosity, and surface tension. Measurements were done by triplicate and independently.

The viscosity was measured with a Discovery HR-3 rotary rheometer (TA Instruments, New Castle, DE, USA), equipped with a Peltier concentric cylinder geometry (DIN Rotor). Each solution (~23 mL) was placed in a stainless-steel cup and evaluated in a shear rate interval from 1.32 × 10^−3^−100 s^−1^ at 25 °C. Viscosity is reported as Pa·s. The surface tension was evaluated at room temperature (~25 °C) with K6 (Krüss, Germany) equipment, based on the Dunöy’s ring method. The surface tension is reported as mN m^−1^. The conductivity was estimated using a 3200-conductivity meter (YSI, Yellow Springs, OH, USA) equipped with an ABS Plastic cell (K = 1.0 cm^−1^, model 3252) and reported as μS. Finally, the density was obtained following the ISO 1183-1:2019 law and calculated according with the Equation (1):

(1) $$\rho_{s}=\frac{m_{pfull}-m_{pempty}}{v}\;$$

where $\rho_{s}$ is the sample’s density, v is the volume, $m_{pfull}$ and $m_{pempty}$ represent the weight of the full and empty pycnometer, respectively. All the experiments were carried out according to the manufacturer’s methodology.

### 2.6. Morphological and Physical Characterization

#### 2.6.1. Morphological Characterization

The morphology of the fibers and membranes was examined by scanning electron microscopy (SEM) with a SM-7800F (JEOL, Akishima, TYO, Japan) microscope. The membranes, collected after 3 h of injection, were analyzed by SEM. A sample of 1 cm^2^ was fixed with carbon tape on an aluminum sample holder and then sputter coated with gold before the analysis. Images were taken at 5 kV at 2000× and 30000× of magnification.

The diameter of the fibers was measured following the methodology reported by García-Hernández et al. (2021) [46], as well as their supplementary material. Briefly, each micrograph (2000×) was changed at 8 bit (grayscale) and saved as TIFF format using ImageJ version 1.51w (Wayne Rasband, National Institutes of Health, Bethesda, MD, USA) [47]. Then, they were segmented using the DiameterJ Segment plugin and processed with the plugin of DiameterJ (V.1-018) [48]. Finally, the diameter distribution (histogram) and average fiber diameter were obtained with Microsoft Excel (2019).

#### 2.6.2. Porosity Evaluation

The porosity evaluation consisted in determining the porosity percentage (%P) and pore size distribution. Porosity was calculated according to the report by Chong et al. (2007) [24] using a gravimetric method with Equations (2) and (3).

(2) $$\%P=\left(1-\frac{membrane\;density\;}{PVA\;density}\right)\times 100$$

(3) $$Membrane\;density=\left(\frac{mass\;of\;membrane}{area\;of\;membrane\times thickness\;of\;membrane}\right)\;$$

Regarding the pore size distribution, this was measured from 2000× SEM images. Briefly, pre-processed at grayscale and thresholded images were used. After that, each image was automatically analyzed using ImageJ/FIJI, version 1.51w (National Institute of Health, Bethesda, MD, USA). Data are reported according to the Feret’s measurements (longest distance between two points along the selection boundary into a hole). The pore size distribution (histogram) and average pore size were obtained.

#### 2.6.3. Thickness

The membranes thickness was estimated with a digital micrometer (H-2780, Mitutoyo, Japan). Ten measurements were taken at random, in an area of 142.5 cm^2^. The results shown are the average of the measurements ± standard deviation [49].

#### 2.6.4. Barrier Properties

Water vapor permeability (WVP) and water vapor transmission rate (WVTR) were measured according to a modification of the thermogravimetric method based on the ASTME-96 as well as following the methodology proposed by Valdespino-León (2020) [50].

For this evaluation, circular samples (51.1 mm of diameter) were closed on a wide-mouth cell filled with DW. Then, the cell was placed on an analytical balance (OHAUS, Parsippany, NJ, USA) to record the cell’s weight change for 4 h and inside a cylindrical capsule with anhydride Drierite desiccant at 37 °C. Data were plotted as weight vs. time and the slope of the curve was calculated (r^2^ < 0.99). The WVTR and WVP were calculated as follows:

(4) $$WVTR=\frac{water\;loss}{time\times area}$$

(5) $$WVP=WVTR\cdot \frac{x}{∆P}\;$$

where the $\frac{water\;loss}{time}$ is the slope. *WVP* was calculated with Equation (5), where *x* is the membrane thickness (m) and ∆P is equal to 6331.5 Pa and corresponds to the vapor pressure difference inside the system

### 2.7. Chemical, Thermal, and Structural Characterization

#### 2.7.1. Fourier-Transform Infrared Spectroscopy (FTIR)

FTIR spectroscopy was performed on a Cary 360 (Agilent Technologies, Santa Clara, CA, USA). Measurements were taken by triplicate with independent samples in a range from 4000 cm^−1^ to 650 cm^−1^. The raw material and each membrane were evaluated and the spectra presented is the average of these measurements. The peaks of interest are associated to the presence of amide I and II in the HC and aromatic rings from the HP and the crystalline and amorphousness-related peaks for the PVA. Data were analyzed on Origin software, Version 8 (OriginLab Corporation, Northampton, MA, USA).

#### 2.7.2. X-ray Diffraction

To study the effects that the manufacturing process has on the polymer’s crystallinity, the polymer and membranes were examined by X-ray diffraction. The granules of PVA were pulverized (30 min) in a coffee grinder (Hamilton Beach, 80350R, Glen Allen, VA, USA), while the HC was used as purchased (powder) and the membranes as obtained. Measurements were done in a diffractometer (Empyrean, Malvern Panalytical, UK) under the following conditions: 45 kV–40 mA, Cu radiation source, filtered to Kα wavelength of 0.154 nm, step size of 0.0263°, and from 5 to 50° in 2θ. Diffractograms (area under the curves) were treated using gaussian deconvolution with Origin software (OriginLab Corporation, Northampton, MA, USA), V.9.8. The degree of crystallinity *C* was calculated as follows:

(6) $$C=\frac{I_{c}}{I_{t}}\;$$

where $I_{c}=\sum I_{p}$, is the sum of the area under the curve of all diffracted peaks (crystalline peaks) $\left(I_{p}\;\right)$; $I_{t}=I_{c}+I_{a}$, is the total intensity (area under the curve, from 5–50°, being $I_{a}$ the intensity of the amorphous component of the sample) [51]. The relative crystallinity ($RC_{m}$) of each membrane (*m*) according to the degree of crystallinity of the pristine PVA, $C_{pva}$, was calculated as follows in Equation (7):

(7) $$RC_{m}=\left(\frac{C_{m}}{C_{pva}}\;\right)\times 100$$

#### 2.7.3. Differential Scanning Calorimetry (DSC)

DSC measurements were done on a DSC 2000 (TA Instruments, New Castle, DE, USA). Briefly, an empty aluminum pan (<10 mg) was used as a reference probe, while 5–6 mg of each material (raw or membrane) was placed in an aluminum standard pan with a hole lid. Samples were heated from 5 to 350 °C at a heating rate of 10 °C min^−1^ in a nitrogen atmosphere (flow rate: 20 mL min^−1^) [52]. Experiments were performed by triplicate with independent samples. In this analysis, denaturation temperature (*T_D_*), decomposition temperature (*T_DS_*), melting temperature (*T_m_*), and glass transition temperature (*T_g_*) are reported.

### 2.8. Biological Characterization

#### 2.8.1. Anti-Inflammatory Activity In Vitro Assessment

The anti-inflammatory activity of the biomembranes was evaluated by the in vitro method of human red blood cell membrane stabilization (HBRC) according to the report by Kumar et al. (2013) [53]. In this assay, erythrocytes are challenged with hemolytic stimuli such as heat. The hemolysis induced on these cells is used as a simple tool to determine the anti-inflammatory properties of materials [54]. These erythrocytes are an effective means for their study since their plasma membrane resembles the lysosomal membrane, so its stabilization implies that the membranes will also protect the lysosomal membrane. In this way, the stabilization of the lysosomal membrane will lead to the inhibition of the release of inflammatory mediators and the consequent inhibition of the inflammation process, avoiding tissue damage [54].

Briefly, a suspension of red blood cells was prepared, combining a sample of fresh blood and sterilized Alsever solution (2% dextrose (J. T. Baker, 10764534), 0.8% sodium citrate (Sigma Aldrich, MO, USA; 1613859), 0.05% citric acid (Sigma Aldrich, MO, USA; 251275), and 0.42% sodium chloride (Sigma Aldrich, MO, USA; S9888) in water in a ratio of 1:1. HBRC were obtained from healthy volunteers following the approved CONBIOÉTICA-09-CEI-002-20190327 (ENCB/CEI/006/2021; ENCB-IPN) protocol. Then, this mixture was centrifuged at 2960 *g* (MetrixLab Dymanica, Velocity 18R, The Netherlands) for 10 min. The cell pack was recovered by decantation and washed four more times with saline solution (0.85%, pH 7.2). Subsequently, with this cell package, a 10% v v^−1^ suspension in isosaline solution was prepared called HRBC [55]. Then, the reaction mixtures were prepared as shown in Table 2.

The reaction mixtures were then subjected to hemolysis by heating in a water bath (Fisher Scientific, Isotemp 102, USA) at 56 °C for 30 min. After this time, they were removed from the bath, cooled at room temperature, and centrifuged at 2220× *g* (MetrixLab Dymanica, Velocity 18R, CDMX, Mexico) for 10 min. Finally, the absorbance of the supernatant at 560 nm was measured in a plate reader (Thermo Fisher Scientific, Multiskan Go, Wattham, MA, USA) and the stabilization % was calculated as shown in Equation (8) (M.G. & Agrawal, 2016) [56]:

(8) $$\%\;stability=\left(\frac{Absorbance\;of\;the\;BC-Absorbance\;of\;the\;Sample}{Absorbance\;of\;the\;BC}\right)\times 100$$

#### 2.8.2. Antimicrobial Activity

Antimicrobial activity was determined using the agar disk diffusion method, according to Çakmak et al., (2020) [57] with some modifications. Previous to antimicrobial analysis, all the samples were sterilized by exposing them to UV light for 10 min on each side. The antimicrobial activity was determined against *Staphylococcus epidermidis* (SE)*, Staphylococcus aureus* (SA)*,* and *Escherichia coli* (EC)*,* because they are reported as the main bacteria responsible for nosocomial infections and/or being frequently isolated from surgical wounds [58,59]. For each microorganism, 100 μL of microorganism solution with a concentration of 10^6^ cells mL^−1^ was spread in petri dishes. Membrane discs were placed in the inoculated Petri dishes and incubated at 37 °C for 24 h. At the end of the incubation time, growth was observed in each petri dish.

### 2.9. Statistical Analysis

Statistical analysis was performed in Sigma Plot V. 12.0, according to one-way analysis of variance for all samples, with at least three independent samples in each experiment. Results are shown as the mean ± standard deviation. Moreover, a Pearson correlation (*p* < 0.05) was carried out to determinate the linear correlation between porosity, thickness, and WVP parameters.

## 3. Results and Discussion

In this work, PVA and HC solutions added with an ethanolic extract of *Hypericum perforatum* (EEHP) at four different concentrations (0, 8, 16, and 32% (*w v*^−1^)) were successfully electrospun to obtain fibers and membranes to be potentially used as wound dressings.

### 3.1. Physicochemical Solutions Characterization

**Figure 2:**
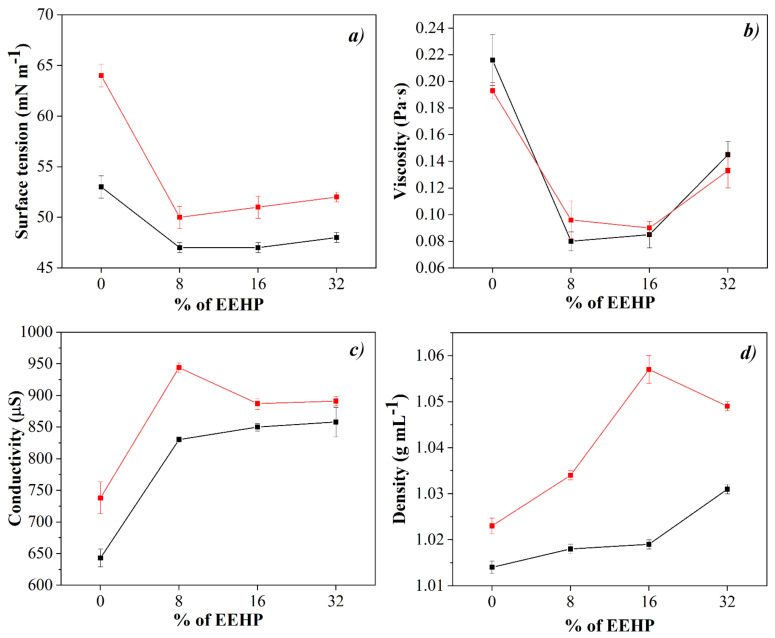
Physicochemical properties of the PVA and PVA/HC solutions added with different concentrations of EEHP. Black line, solutions without HC (6-0-0, 6-0-8, 6-0-16, and 6-0-32), and in red line, solutions with HC (6-5-0, 6-5-8, 6-5-16, and 6-5-32).

Figure 2 shows the physicochemical properties of the solutions used to fabricate the electrospun membranes made up of PVA, HC, and EEHP. The addition of EEHP to the solutions to be electrospinnable decreased the viscosity up to a minimum at 16%, increasing at higher extract concentrations. The presence of HC did not change the viscosity behavior among samples. Surface tension values also decreased at higher EEHP concentrations. This behavior could be due to the higher surface tension of water (72.8 mN m^−1^) used as solvent media for the HC, while the ethanol is 22.8 mN m^−1^ used for the extract preparation. Moreover, it was possible to observe that the surface tension is not dependent on the EEHP concentration as there are no significant differences between the solutions (*p* < 0.05).

Regarding density and conductivity, they show an increase (*p* < 0.05) according to the EEHP concentration in the PVA/EEHP solutions and a more notable increase when the HC solution is added. The augment in density can be related to the increase in total solids in each solution, while the increase in conductivity can be attributed to the intrinsic polyelectronic behavior of the hydrolyzed collagen polypeptides [25]. In this sense, it has been reported that a higher conductivity favors the fibers formation and can reduce their diameter [60].

### 3.2. Fabrication and Microstructural Characterization of the Electrospun Membranes

**Figure 3:**
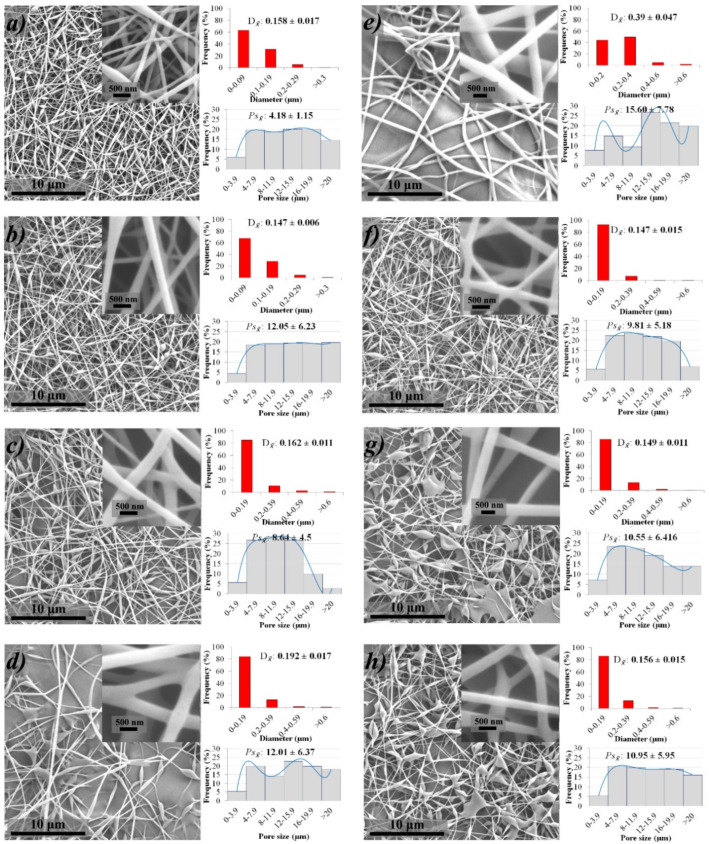
Morphological characterization by SEM of fibers (inserted SEM images at 30000×) and biomembranes (SEM images at 2000× fabricated by electrospinning technique from PVA/HC/EEHP: (**a**) 6-0-0, (**b**) 6-0-8, (**c**) 6-0-16, (**d**) 6-0-32, (**e**) 6-5-0, (**f**) 6-5-8, (**g**) 6-5-16, and (**h**) 6-5-32. Fiber diameter (mean: D*_x_* (µm) and distribution histogram (red)) and pore size (mean: Ps*_x_* (µm), and distribution histogram (grey)). Nomenclature: % PVA (6)-% hydrolyzed collagen (0, 5)-% EEHP (0, 8, 16, 32).

Figure 3 shows the microstructural arrangement of the biomembrane (SEM micrographs at 2000×) diameter and pore size distributions. Regarding the microstructural arrangement, the 6-0-0 (Figure 3a) sample is made up of only fibers, while all the rest that contain EEPH at different concentrations (8, 16, and 32% (*w v*^−1^)) presented a combination of fibers/film character with a higher incidence of film as the concentration of the extract increased (Figure 3b–d). This phenomenon became more evident in all the membranes added with the HC (Figure 3e–h). The presence of beads was also observed at a higher concentration of extract and collagen addition. Authors such as [61] report the presence of beads attributed to the low viscosity of the solutions, as happens in our case, when compared between solutions without EEHP (6-0-0 and 6-5-0) vs. with EEHP, and that is also related to a larger film structure [61].

Although the structure and morphology of the final products depend on the synergistic effect of solution parameters and electrostatic forces (viscosity, surface tension, concentration, dielectric properties of the spinning solution, and acceleration), the viscosity is considered the dominant parameter which influences them, as well as spinnability (whether solutions will be electrospun), which also depends on the ability of the polymer solution to create relatively stable jets [60,62]. As reported, solutions with lower viscosity (<0.1 Pa·s) can lead to the disruption of polymer filaments and can produce polymer droplets or beads; here surface tension is the dominant influence on fiber morphology, while at higher concentrations (>2 Pa·s) polymer extrusion is impossible. Thus, the minimum required viscosity corresponds to a certain polymer concentration in the solution to be electrospun and varies depending on the molecular weight of the polymer and the nature of the solvent used, which, according to [62,63] the optimal viscosity of a spinning solution is 0.1–2 Pa·s, which does not break the jet and travels as a jet to the grounded target [62,63]. Nevertheless, as in this work, although most of the electrospun solutions are within the range, the fibers show beads along their surface, which according to [64] can be inhibited by increasing the viscosity of the solutions, that is, the concentration of the polymer [64]. However, as also has been reported, the presence of beads as we have on there are not affected by the fabricated membranes, since the structure has been proved as an effective medium to load bioactive compounds, encouraging benefits in suitable drug release [64,65].

About the diameter (D), this varies from 140–390 nm with a monodisperse behavior when the EEHP concentration increases, which at the highest concentration (32%) the 80% of the fibers have a diameter of 190 nm. These values are lower than those reported by [46] on PVA-based and PVA/HC membranes, due to the ambient conditions (humidity and temperature) in this work being controlled. Moreover, according to [60] the environmental conditions affect the characteristics of the fibers formed, finding that at higher temperature the humidity is reduced and the solvent evaporates faster, obtaining fibers with smaller diameters.

Concerning pore size (Figure 3), all the samples presented a multimodal distribution with average sizes from 4–16 µm. This behavior is attributed to the random deposition of the fibers on the collector [24]. However, according to various authors, all membranes have optimal pore size to be used as wound dressings, since it is estimated that neovascularization and cell growth (e.g., fibroblasts) can be generated with pore sizes between 3–350 µm [24,66,67,68]. Indeed, according to previous reports our biomembranes showed better values than some commercial or published ones [49,56,69].

**Figure 4:**
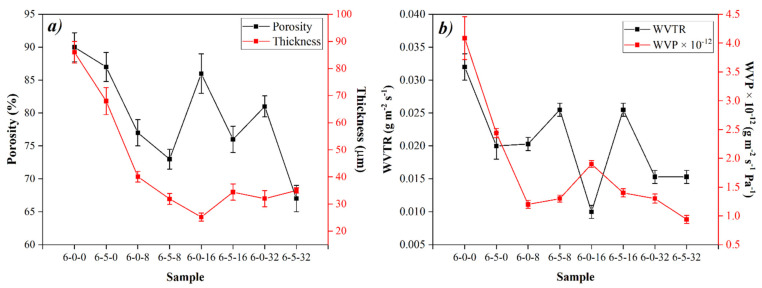
(**a**) Thickness and porosity of the membranes and (**b**) barrier properties of the membranes.

Another parameter of great importance to define the applicability of biomembranes to be used in the biomedical field (e.g., wound dressings, scaffolds, etc.,) for skin reconstruction is porosity, which must simulate the extracellular matrix (ECM) with a porosity range between 60–90% to allow cell growth, interaction, and proliferation [24,66]. Given these values and according to the report in Figure 4a, we can infer that our materials are optimal devices to treat wounds due to all of them presenting porosity values between 67–90%, variation attributed to the microstructural arrangement of the membranes, since while sample 6-0-0 with the highest percentage of porosity is composed only of fibers (fibers with few beads) the samples added with HC (6-5-0), EEHP (6-0-8, 6-0-16 and 6-0-32), or both (6-5-8, 6-5-16 and 6-5-32) presented a greater number of beads along its surface, decreasing the free volume between the fibers. Moreover, as shown in Figure 3 the increase in EEHP concentration and the presence of HC increases film formation, also decreases free volume and, therefore, porosity.

Regarding thickness (Figure 4a), it was found that all the membranes added with EEHP presented significant differences (*p* < 0.05) compared with the samples of only-PVA (6-0-0) and PVA/HC (6-5-0) [70]. This behavior can be related with the film microstructural arrangement, where the fibers are agglomerated and thus the free volume decreases, as well as the porosity and thickness values [71].

### 3.3. Barrier Properties: Water Vapor Permeability (WVP) and Water Vapor Transmission Rate (WVTR)

WVP and WVTR play a very important role when membranes want to be applied in the biomedical field for wound protection. As reported by [72], tissue regeneration or repair is only carried out when the biomembrane has suitable conditions to allow the release of liquid and/or moisture without dehydrating the treated wound [69]. Figure 4b reports the barrier properties of EEHP-added biomembranes with and without hydrolyzed collagen. Here, the highest WVP values were observed in the membranes without EEHP (6-0-0 and 6-5-0), while the membranes added with EEHP presented values between 0.9 to 1.9 × 10^−12^ g·m^−1^·s^−1^·Pa^−1^. Additionally, as seen in the same figure, the values did not show significant differences between them (*p* < 0.05). However, it can be argued that the increase in the extract concentration limits or decreases the WVP and WVTR since it has an effect on the materials’ porosity and therefore on the barrier properties, as seen in Figure 4. According to Pearson’s correlation, there is a significant and positive relationship (*p* < 0.05) between thickness vs. porosity (CC:0.813, *p* = 0.0141), thickness vs. WVTR (CC:0.959, *p* = 0.000171), and porosity vs. WVTR (CC:0.81, *p* = 0.0149).

Regarding WVTR, all the EEHP-added membranes show significant differences (*p* < 0.05) when compared with the 6-0-0 and 6-5-0; behavior attributed to the presence of the extract, no matter the concentration or hydrolyzed collagen addition. However, as has been reported, an ideal membrane to be used in the biomedical field should have WVTR values from 0.023–0.03 g·m^−2^·s^−1^ [73]. In this sense, samples 6-5-0, 6-0-8, 6-5-8, and 6-5-16 have adequate values or closest values to the ideal; nevertheless, all membranes present better WVP and WVTR values than some commercial and/or even reported materials WVP: high-density polyethylene (0.24 × 10^−12^ g·s^−1^·m^−1^·Pa^−1^), low-density polyethylene (0.73 × 10^−12^ g·s^−1^·m^−1^·Pa^−1^), and polypropylene (0.49 × 10^−12^ g·s^−1^·m^−1^·Pa^−1^) [74]; WVTR: PCL/gelatin (0.0056 g·m^−2^·s^−1^) and polyurethane (0.0046 g·m^−2^·s^−1^ and 0.0093 g·m^−2^·s^−1^), chitosan (0.01565 g·m^−2^·s^−1^) [75], or PCL (0.017 g·m^−2^·s^−1^) [76], which is advantageous for the desired application.

### 3.4. Chemical, Structural, and Thermal Characterization

Fourier-Transform Infrared Spectroscopy (FT-IR)

**Figure 5:**
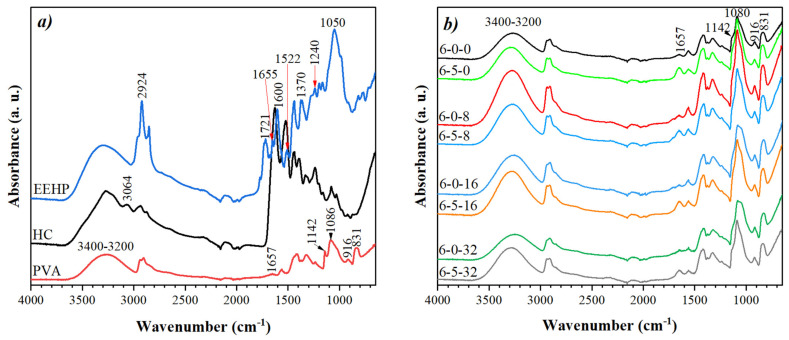
FTIR spectrum of (**a**) raw materials and (**b**) membranes obtained by electrospinning with EEHP at different concentrations (0, 8, 16, 32) and with and without hydrolyzed collagen (5%).

Figure 5a shows the IR spectra of the raw materials, PVA as granules, hydrolyzed collagen as powder, and the ethanolic extract of *Hypericum perforatum* after drying. In the PVA spectrum (Figure 5a, red line), several characteristic bands were located at 3400–3200 cm^−1^ due to the stretching of OH groups at 2938 cm^−1^ and another at 2907 cm^−1^ due to the asymmetric and symmetric stretching of CH_2_, respectively; another peak with low intensity at 1657 cm^−1^ attributed to C=C, a band at 1418 cm^−1^ for the CH bending, CH_2_ stretching and -OH bending modes; a peak at 1375 cm^−1^ due to the wagging of CH_2_, and at 1325 cm^−1^ due to the bending of OH and CH. The last peaks, below 1000 cm^–1^, at 916 and 831 cm^−1^, correspond to the bending and rocking of the CH_2_ groups [77,78,79]. However, the most important bands related to the microstructural arrangement are the bands at 1142 and 1086 cm^–1^; the first one associated with the stretching of CO from the PVA crystal sequence and the second one attributed to CC stretching and OH bending of the amorphous PVA sequence.

Regarding hydrolyzed collagen (Figure 5a, black line), the amide B band was found at 3064 cm^−1^ and is associated with the CH stretching, while the band at 2963 cm^−1^ corresponds to the asymmetric stretching of CH_2_. The amide I band, with characteristic peaks at frequencies between 1600–1700, presented a narrow peak at 1600 cm^−1^, which is mainly associated with the stretching of the carbonyl group (C=O). Regarding amide II and III, their peaks were found at 1522 and 1236 cm^−1^, respectively, representing flexural vibrations of N–H coupled with stretching vibrations of C–N. Another characteristic band at 1333 cm^−1^ is attributed to the bending of CH_2_ [77,80]. Finally, it is stated that the band at 1450 cm^−1^ corresponds to the vibrations of the pyrrolidine rings of proline and hydroxyproline [81]. These results are comparable with non-commercial materials reported in the literature.

The spectrum of the ethanolic extract of *Hypericum perforatum* is also presented in Figure 5a (blue line). EEHP is composed by a wide variety of secondary metabolites such as naphthodiantrones, phloroglucinols, flavonoids, terpenes, phenolic compounds, xanthones, saponins, etc. In this sense, the IR spectrum shows the presence of a broad band corresponding to the stretching vibrations of the OH groups between 3400–3200 cm^−1^ due to the presence of hyperforin, alcohols, phenols, carbohydrates, or water. Other peaks attributed to the presence of two of the most important metabolites of this extract, hyperforin and hypericin, occur at 2924 cm^−1^ due to the stretching of CH, at 1721 cm^−1^ due to the vibration of C=O of the ketone group, at 1655 cm^−1^ due to the stretching of C=C, at 1598 cm^−1^ due to the stretching vibration of the carbonyl group, as well as the stretching of C-C and deformation of H-O-C [82,83]. In this spectrum, and due to the presence of other components in the extract, a peak at 1370 cm^−1^ due to the aromatic stretching of C-C is also observed and due to the deformation in the plane of the methyl group and phenolic -OH groups; a peak at 1240 cm^−1^ by the stretching vibration of the C-C bond, and by in-plane deformation of the C-C-H and H-O-C bonds; a low intensity peak at 1200 cm^−1^ related to the C-O phenolic groups; and bands between 700–1050 cm^−1^ attributed to in-plane and out-of-plane deformation vibrations, as well as the stretching vibration of aromatic rings, present in a variety of secondary metabolites [3,42,84,85].

The FT-IR spectra of the electrospun membranes obtained by electrospinning with the addition of EEHP at different concentrations (0, 8, 16, 32%) and added or not with HC are shown in Figure 5b. These spectra present some modifications when compared with the PVA pattern as raw material. First, the broad peak around 3400–3200 cm^−1^, associated with the OH stretching present greater intensity in the membranes of 6-0-0 and 6-5-0, but even more intensity in the EEHP-added membranes. This behavior can be related with the increment of the stretching vibrations due to the hydroxyl groups of water in the PVA chains [86] and the –NH_2_ and -OH groups of the HC and/or the -OH groups from hypericin, hyperforin, or other metabolites from the ethanolic extract, which generates good compatibility in the blended membranes.

The band at 1142 cm^−1^, attributed to the polymer crystallinity, decreased notably in all cases, changing to a broader and more intense peak at 1087 cm^−1^. This behavior could be related to the crystallinity loss or amorphousness of the PVA after processing. In this sense, it is confirmed that the electrospinning process decreases the materials’ crystallinity [77]. Around these wavenumbers another important modification occurs: the peak at 1050 cm^−1^ is better defined when the concentration of EEHP increases, which is associated with the presence of EEHP and therefore with stretching vibrations of the aromatic rings of the secondary metabolites. However, when HC is added (EEHP decreases, Table 1) this peak tends to disappear.

Regarding the peak at 1657 cm^−1^, in the region of amide I, the samples added with EEHP showed a greater peak, compared with the only-PVA (6-0-0), and even more so in those added with collagen peptides (6-5-8, 6-5-16, 6-5-32; Figure 3c), confirming the presence of these components, since said peak as mentioned is attributed to the stretching of CH into hypericin and hyperforin and to the characteristic peak region of Amide I. Other intensity changes in the peak at 831 cm^−1^ indicate greater stretching vibration due to the presence of benzene rings, which even suggests a good compatibility between the components used [77,86].

In the same biomembrane spectra, some peaks identified with certainty are related to the characteristic peaks of the main EEHP compounds. This may be due to the low concentration of the metabolites in the membranes or these are not detectable due to the overlapping with other main PVA or the HC peaks, given the compatibility of the formulation. Other authors have reported a similar behavior in fibers fabricated from polyvinylpyrrolidone added with hemp extract [3,87].

### 3.5. X-ray Diffraction (XRD)

**Figure 6:**
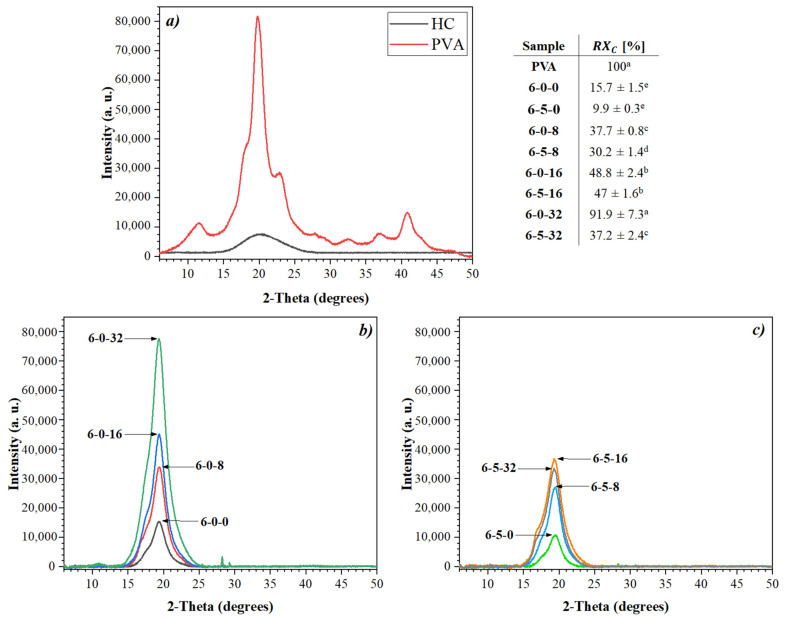
X-ray diffraction patterns: (**a**) PVA and HC (**b**,**c**) biomembranes produced by electrospinning with PVA/HC/EEHP. (Nomenclature: % PVA (6)—% hydrolyzed collagen (0, 5)—% EEHP (0, 8, 16, 32)). *RX*—relative crystallinity percentage. In the table, same letters in the same column indicates no significant difference between the data (*p* < 0.05). Nomenclature: % PVA (6)—% hydrolyzed collagen (0, 5)—% EEHP (0, 8, 16, 32).

Figure 6 shows the XRD patterns of PVA and hydrolyzed collagen, as well as the electrospun membranes with and without HC (Figure 6a,b). The PVA XRD pattern (Figure 6a, PVA) have many well-defined peaks at 11.1°, 19.5°, 23.4°, 28.1°, 32.7°, 37, and 41.5° in 2θ. These peaks are associated with the pseudo-orthorhombic or monoclinic crystalline cell of PVA [88]. In the same figure, the hydrolyzed collagen pattern shows a broad peak at 2θ = 20°, associated with an amorphous structure [89]. In contrast, the XRD patterns of the membranes (Figure 6b,c) only show a peak at 2θ = 19.5°, suggesting a crystallinity degradation phenomenon due to the addition of HC and the electrospinning process [90], which is reversed by the incorporation of EEHP as observed in the relative crystallinity values in Table in Figure 6.

In general, as has been reported before, the loss of crystallinity is due to a rapid drying process that occurs during the fiber’s fabrication, since the diffusion and growth of the polymer chains is limited to forming crystals [90,91]. These results are congruent with those reported by Pourhojat et al. (2017) [3] on electrospun PCL membranes, where the higher concentration of *Hypericum perforatum* the higher crystallinity. Likewise, the increase in crystallinity as a result of the addition of EEHP may be due to the alignment of the polymeric chains, facilitating the formation of molecular bonds between their hydrogen atoms; a hypothesis supported by the infrared spectroscopy results previously presented.

### 3.6. Differential Scanning Calorimetry

**Figure 7:**
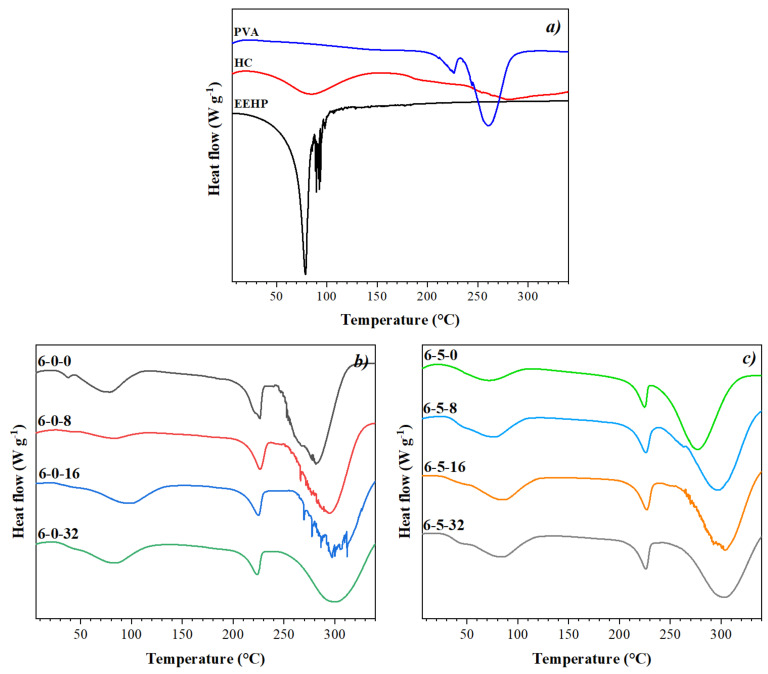
DSC thermograms of (**a**) the raw materials: hydrolyzed collagen (HC), ethanolic extract of *Hypericum perforatum* (EEHP), and PVA and (**b**,**c**) membranes obtained by electrospinning with PVA/HC/EEHP. (Nomenclature: % PVA (6)-% hydrolyzed collagen (0, 5)-% EEHP (0, 8, 16, 32)).

Figure 7 shows the DSC results for the raw materials (Figure 7a) and the biomembranes of PVA and EEHP with and without HC (Figure 7b and Figure 7c, respectively). From the PVA thermogram, two endothermic peaks can be identified: the first one at 225 °C attributed to the melting temperature (*T_m_*) and the second one around 260–280 °C due to the material decomposition temperature (*T_Ds_*). About the HC, it is possible to observe that this shows an endothermic peak at 84 °C due to the denaturation or dehydration temperature (*T_D_*), which represents the destabilization of H bonds and water molecules in the protein elimination. Authors such as Foltran et al. (2008) [92] reported the values of *T_D_* from 82.35 °C to 85 °C, the difference being the degree of hydration not the source of collagen [92]. Hydrolyzed collagen also presented a phase transition at higher temperatures (188 °C), which corresponds to the decomposition temperature (*T_Ds_*) [93] as a result of a chemical reaction that results in thermolysis (pyrolysis or carbonization) [94]. Meanwhile, the EEHP thermogram shows an endothermic peak at 80 °C due to the decomposition temperature (*T_Ds_*).

Regarding the thermograms of the membranes, they presented some variations in the baseline close to 45 °C (Figure 5b,c); a phenomenon associated to the PVA glass transition temperature (*Tg*) due to its amorphous structure as shown in FT-IR and XRD analysis. Additionally, the DSC image shows a broad peak from 60–90 °C, which can be related with the solvent evaporation as has been reported before on chitosan/PVA fibers [90].

These thermograms also show an endothermic peak at 225 °C as previously reported by Ding et al., (2010) [95], which corresponds to the melting point (*T_m_*) of PVA, [95] and another peak around 280–300 °C attributed to its decomposition temperature [96], which actually defers with the PVA granules and can be related to the membranes’ fibers structural arrangement. However, as can also be elucidated, the no EEHP-added membranes (6-0-0 and 6-5-0) presented this endothermic peak with a maximum at 278 °C, while the *T_Ds_* of the EEHP-added presented at around 290–300 °C by increasing the *T_Ds_* when the EEHP concentration increases. This behavior could be related to the crystallinity, as stronger molecule interactions between the components require higher energy (temperature) to decompose the materials.

### 3.7. Biological Characterization

Biological in vitro characterization was carried out to know their anti-inflammatory and antimicrobial capacity and thus verify their applicability as a biomedical device to treat skin injuries.

Anti-inflammatory activity

**Figure 8:**
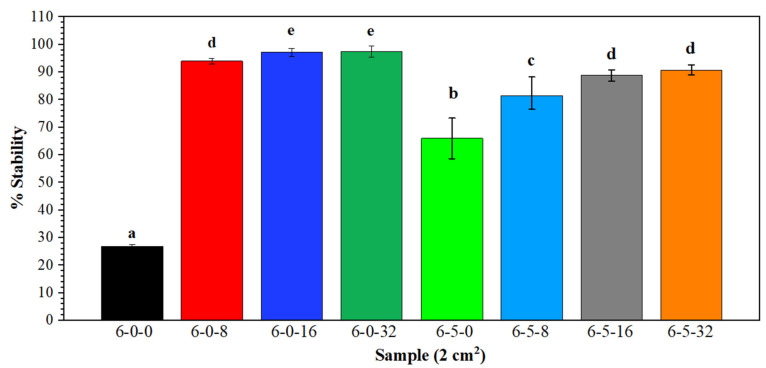
Anti-inflammatory activity of PVA/HC/EEHP electrospun membranes (Nomenclature: % PVA (6)—% hydrolyzed collagen (0, 5)—% EEHP (0, 8, 16, 32)).

Figure 8 shows the stability percentage of red blood cell protection, which is translated as the anti-inflammatory activity of the biomembranes according to the basic principle of the HRBC method used to determine the activity. According to the statistical analysis, there is no significant difference (*p* < 0.05) between samples 6-0-8, 6-5-16, and 6-5-32. Furthermore, it can be seen that the 6-5-0 membrane provides 66% protection, attributed to HC, while the membranes 6-0-8, 6-0-16, and 6-0-32 slightly increase the stability according to the concentration of EEHP, from 93% for the less concentrated sample (6-0-8) to 98% for the most concentrated (6-0-32).

Regarding the membranes with the three components, 6-5-8, 6-5-16, and 6-5-32, these have less protection, 81%, 88%, and 90%, respectively, indicating that: (1) at higher EEHP concentration there is greater protection and (2), although the HC favor the anti-inflammatory activity of the biomembranes, the greater protective effect is attributed to the presence of the extract due to diminish when added HC and to the less used EEHP into the solutions, as reported in Table 1.

The anti-inflammatory activity is mainly attributed to the synergistic action of the secondary metabolites of the extract, naphthodiantrones, phenolic compounds, and flavonoids [97,98].

Antimicrobial activity

**Figure 9:**
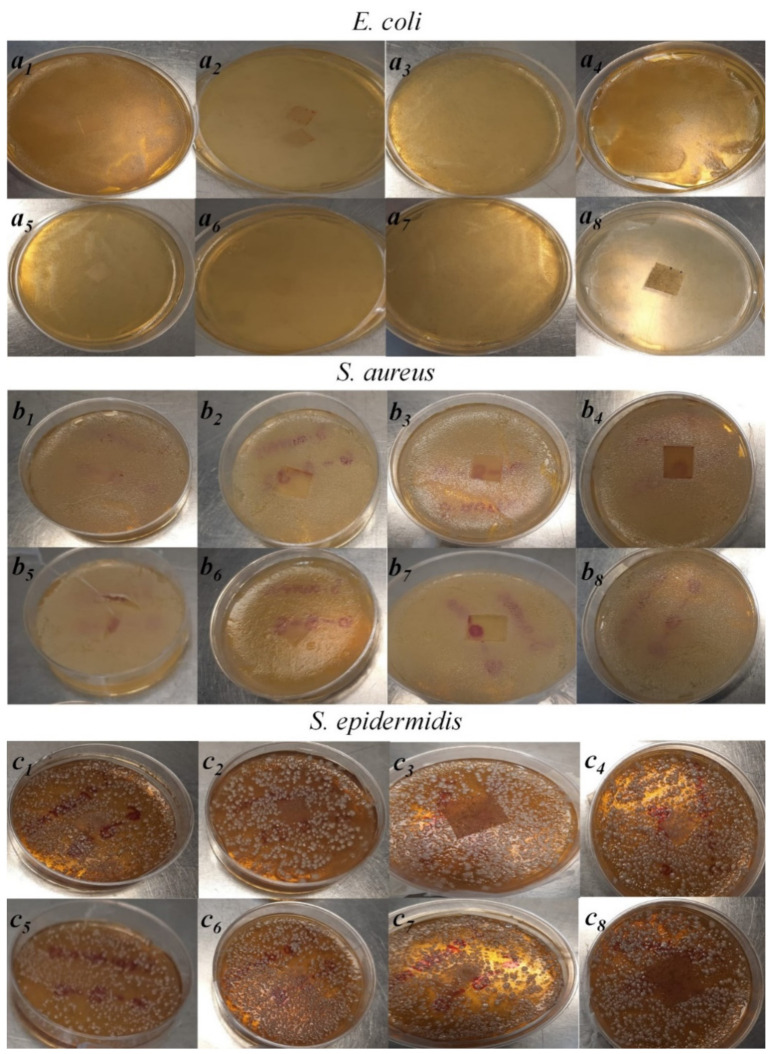
Antimicrobial activity of PVA/HC/EEHP electrospun membranes (Nomenclature: % PVA (6)—% hydrolyzed collagen (0, 5)—% EEHP (0, 8, 16, 32)).

Figure 9 shows the antimicrobial activity of the membranes against *E. coli* (Figure 9a), *S. aureus* (Figure 9b), and *S. epidermis* (Figure 9c). Any membrane showed antimicrobial activity against *E. coli* (Figure 9a), regardless the presence of HC or the concentration of EEHP. For the EEHP-only samples, 6-0-8, 6-0-16, and 6-0-32 presented activities against *S. aureus* and *S. epidermis* (Figure 9b and c, respectively), 6-0-16 being the one with the highest activity against both microorganisms. The 6-5-16 membrane showed inhibition against *S. aureus*, however not against *S. epidermidis*, showing growth on the membrane.

In all cases, a higher effect was observed against *S. aureus*, where there was no growth of microorganisms on the membranes or their periphery, while with *S. epidermidis* a low growth was observed on the edges of the membranes. It should be noted that the membrane did not show extract diffusion, since the inhibition was only observed in the membrane without generating an inhibition halo.

Some studies have reported that the extract of *Hypericum perforatum* only has antimicrobial activity against Gram positive bacteria [30]. One cause of the extract’s low or null activity against *E. coli* is its characteristic outer membrane of Gram-negative bacteria. This membrane has porins, which have been shown to have a retarding capacity in the access of antibiotics to the bacteria interior [99,100].

## 4. Conclusions

PVA/HC/EEHP membranes were successfully fabricated by the electrospinning technique. Furthermore, fibers with diameters between 140–390 nm were obtained, with morphology dependence mainly on the polymer/solvent systems and their physicochemical properties. XRD, FT-IR, and DSC analysis corroborated a general loss of crystallinity when compared with the PVA as raw material but higher crystallinity according to the EEHP concentration. These membranes are a potential option to be used in the biomedical field to treat skin injuries due to their composition, optimal porosity measurements (porosity: up to 70%, pore size: 4–16 µm), and barrier properties (WVP: 0.94–4.085 g·m^−1^·s^−1^·Pa^−1^), which are close to or even better than other reported or commercial products to be applied as potential biomedical devices to treat skin injuries.

## Figures and Tables

**Table 1:** Nomenclature of the fabricated membranes.

Concentration [%] *			PVA:PepColl:EEHP Ratio (*v*:*v*:*v*)	Nomenclature
PVA	HC	EEHP		
6	0	0	10:0:1 **	6-0-0
6	5	0	10:1:0	6-5-0
6	0	8	10:0:1	6-0-8
6	5	8	10:0.5:0.5	6-5-8
6	0	16	10:0:1	6-0-16
6	5	16	10:0.5:0.5	6-5-16
6	0	32	10:0:1	6-0-32
6	5	32	10:0.5:0.5	6-5-32

PVA—polyvinyl alcohol, HC—hydrolyzed collagen, *EEHP*—ethanolic extract of *Hypericum perforatum*. * PVA(%)/HC(%)/EEHP(%). ** EtOH—ethanol at 96%.

**Table 2:** Reaction tubes for in vitro anti-inflammatory evaluation.

Tube	Solutions
Blank	0.5 mL of NaCl + 0.25 mL of PBS + 0.375 of DW
Blood control (BC)	0.5 mL of NaCl + 0.25 mL of PBS + 0.125 mL of HRBC + 0.25 mL of isosaline
Positive control	0.5 mL of NaCl + 0.25 mL of PBS + 0.125 mL of HRBC + 0.25 mL of diclofenac
Sample	0.5 mL of NaCl + 0.25 mL of PBS + 0.125 mL of HRBC + 0.25 mL of isosaline solution + membrane

The solution concentration was as follows: NaCl at 0.25% pv^−1^, PBS (Sigma Aldrich, MO, USA; P5493) at 0.15 M (pH 7.4), and diclofenac (Sigma Aldrich, D6899) at 5 mg mL^−1^.The membrane had an area of 2 cm^2^.

## Data Availability

The data presented in this study are available on request from the corresponding author.
